# Online re-examination of postgraduate medical students during the COVID-19 pandemic

**DOI:** 10.1186/s12909-022-03100-8

**Published:** 2022-01-18

**Authors:** Shixian Gu, Wenqing Yuan, Aijing Zhang, Gang Huo, Mengyun Jiang, Jiangli Han, Ning Shen

**Affiliations:** 1 Department of Education, Department of Cardiology, Peking University Third Hospital, 38 Xueyuan Road, Haidian District, 100191 Beijing, China; 2 Department of Cardiology, Peking University Third Hospital, 38 Xueyuan Road, Haidian District, 100191 Beijing, China; 3 Department of Pulmonary and Critical Care Medicine, Peking University Third Hospital, 38 Xueyuan Road, Haidian District, 100191 Beijing, China

**Keywords:** Education, Medical, Postgraduate, Online re-examination, COVID-19

## Abstract

**Background:**

Postgraduate entrance examination (the Unified National Graduate Entrance Examination) is the major way for Chinese medical undergraduate student to apply for postgraduate studies. It consists of two stages: the preliminary basic written test and the re-examination in form of both written tests and interviews. With the spread of COVID-19, the traditional on-site re-examination of postgraduates must be changed to online re-examination. By comparing the re-examination process and admission results of online and on-site re-examination, we studied the feasibility of online re-examination for postgraduates and measures to improve it.

**Methods:**

This was a retrospective cohort study using data from the Unified National Graduate Entrance Examination. Our sample population was the applicants to Peking University Third Hospital (PUTH) who completed re-examinations. In total, 281 records were successively selected from March 2017 to May 2020. By comparing the re-examination process and admission results of the 2020 online re-examination with those of the 2017-2019 on-site re-examinations, we analyzed the process, difficulties and improvement of online re-examination.

**Results:**

A total of 281 subjects were included, of whom 77.9% completed an on-site re-examination in 2017-2019 and 22.1% completed the 2020 online re-examination. In the on-site re-examinations, 70.8% of the students were admitted, and in the online re-examination, 74.2% of the students were admitted. There were no significant differences between the students who completed on-site and online re-examinations in terms of gender, recent graduation, cultivation type, graduate from a key university, and admission (*P*>0.05). The on-site and online re-examination results were very similar among the admitted students. The multivariable logistic regression analysis showed that online re-examination had no effect on student admissions. Students seeking professional degree were less likely to be admitted than those seeking academic degree, and those with a better standardized rank in medicine and a better standardized rank of re-examination score were more likely to be admitted.

**Conclusions:**

The online re-examination implemented in 2020 during the COVID-19 pandemic achieved the same selective effect as on-site re-examination. Effective time management, a standardized test question template, well-trained staff and effective technology are the keys to success.

## Background

In late 2019, multiple cases of pneumonia of unknown etiology were observed in the city of Wuhan in Hubei Province in China. Soon, following genomic sequencing, it was found that these cases were caused by a novel virus called severe acute respiratory syndrome coronavirus 2 (SARS-CoV-2), also known as 2019 novel coronavirus (2019-nCoV) [1–4]. The infection caused by the virus, termed coronavirus disease 2019 (COVID-19), spread throughout the world, leading the World Health Organization to declare a global pandemic on March 11, 2020 [1, 5]. As of June 26, 2020, more than 9.4 million confirmed cases of COVID-19 had been reported, with more than 484,000 attributable deaths worldwide [6].

During the current pandemic, most countries have implemented responses to contain the COVID-19 pandemic using different strategies, such as contact tracing and self-quarantine; the establishment of health system infrastructures to treat severely infected patients; the reduction or prohibition of mass gatherings; and the encouragement of the application of hygienic measures, such as physical distancing, respiratory etiquette and frequent hand washing. In the absence of a COVID-19 treatment, the application of protective measures will potentially prevent the population from acquiring the disease and reduce disease dissemination [7, 8].

China has gone through rounds of reform for medical education. Medical postgraduate degrees in China have been divided into two types based on fundamental training goals: the professional degree and the academic degree. The fundamental training goal for academic degree is to conduct scientific research, while the primary training goal for professional degree is to achieve competent clinical skills. The main culturing model for both medical postgraduate degrees now is 5 years of undergraduate medical education and then taking the Unified National Graduate Entrance Examination for a 3 years of professional master degree [9]. The Unified National Graduate Entrance Examination is the selection test organized by the Ministry of Education of the People’s Republic of China. It is an important way to select high-level advanced medical students in China, and consist of two main procedures: the preliminary test and the re-examination. The preliminary examination is a national unified examination testing basic knowledge with a total score of 500 points. It contains tests of subject 1 (Politics) and subject 2 (General English). The re-examination test comprehensive quality and ability including knowledge utilizing, communication skills, resilience, etc. The procedure usually consists of written test followed by an interview with a panel of experts. In the written test, professional English, professional knowledge, clinical skills, scientific research skills and other competencies. The total score is calculated based on the preliminary examination score and the re-examination score, each accounting for 50% of the total score, and the final admission decision is made based on the total score. In the past, the re-examination was completed on site. However, in the new situation of the COVID-19 pandemic, although the pandemic is well controlled, it still involves risks in the traditional way of on-site examination. The development of online interview softwares, such as Webex, Tecent, etc., have advanced by leaps and bounds. Various functions suitable for online exams, such as waiting room and real-name system were also developed. Thus, after comprehensive consideration and evaluation, the re-examination of postgraduate applicants in 2020 was conducted online.

We were faced with the problem of how to convert on-site activities to online activities and make the examination as fair as it previously had been. Over the past few decades, the application of web-based learning and teaching tools has increased rapidly. It is well known that performance based on online courses is as good as that based on traditional on-site methods [10–16]. However, evidence whether online and on-site assessment are comparable is limited [17, 18]. Under the great impact of COVID-19, the demand for online entrance exams is growing with its effectiveness remains to be studied. It is a new challenge to organize the re-examination of postgraduate student applicants based on the principles of security, fairness and scientific quality. The 2020 online re-examination was well organized, and it was compared with previous on-site re-examinations. In addition, the process, feasibility, difficulties of the online re-examination of postgraduates were measured, and measures for improvement were considered.

## Methods

### Study design

We conducted a retrospective cohort study with data from the Unified National Graduate Entrance Examination. Our sample population was the applicants to Peking University Third Hospital (PUTH) who completed re-examinations. A total of 281 records were successively selected from March 2017 to May 2020. By comparing the re-examination process and admission results of the 2020 online re-examination and 2017-2019 on-site re-examinations, we analyzed the process, feasibility, difficulties and improvement of online re-examination. This retrospective study had been reviewed and approved by the Peking University Third Hospital Medical Science Research Ethics Committee (No. IRB00006761-M2020476). All methods were carried out in accordance with relevant guidelines and regulations. We confirmed that informed consent was obtained from all subjects or, if subjects were under 16, from a parent and/or legal guardian.

We collected data on factors such as age, gender, recent graduation, graduate from a key university, cultivation type, preliminary examination scores and re-examination score. The cultivation type was categorized as either professional degree or academic degree. Preliminary examination scores were collected for Subject 1, Subject 2 and comprehensive medicine from the Unified National Graduate Entrance Examination. The total scores of Subject 1 and Subject 2 are both 100, and the total score of comprehensive medicine is 300. The re-examination items included tests of comprehensive quality, English listening, English speaking, professional English, basic knowledge, professional knowledge, clinical skills and scientific research skills and other competencies. The total score was calculated based on the score of the preliminary examination and the score of the re-examination, each accounting for 50%, and the final admission decision was made based on the total score. The purpose of postgraduate re-examination is to select medical students with excellent comprehensive quality to pursue further study for a master’s degree. This paper was reported in accordance with the STROBE statement.

### On-site re-examination plan

**Fig. 1:**
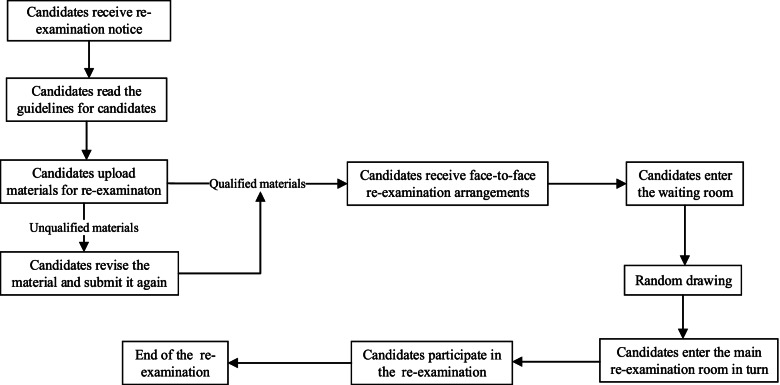
Flow chart of the on-site re-examination in PUTH

 According to the requirements of the Peking University Health Science Center (PKUHSC), the admission-application-ratio for on-site re-examination was 1:1.5, and PUTH determined the list of candidates for re-examination according to their preliminary scores, ranking students from high to low. Candidates for re-examination had to take tests of comprehensive quality, English listening, English speaking, professional English, basic knowledge, professional knowledge, clinical skills and scientific research skills and other comprehensive assessments on site. The total score was calculated based on the score of the preliminary examination and the score of the re-examination, each accounting for 50%, and the final admission decision was made according to the total score. Figure 1 is the flow chart of the on-site re-examination in PUTH.

### Online re-examination plan

We designed online interview with structured standards of operations for the online re-examination in 2020. 117 standardized questions were developed by 97 experts including tests of English, professional knowledge, clinical skills and scientific research skills, comprehensive quality and examination of application materials. In the English listening test, the supervisor read a paragraph in English, and the applicant translated it. Some of the test content was displayed to applicants through screen sharing, and candidates answered the questions. Some of the test content was asked by the examiner online, and the candidates answered immediately. In addition, the supervisor also performed a comprehensive evaluation of the candidates’ application materials, and certain content which was difficult to evaluate through video conferences was not included.

**Fig. 2:**
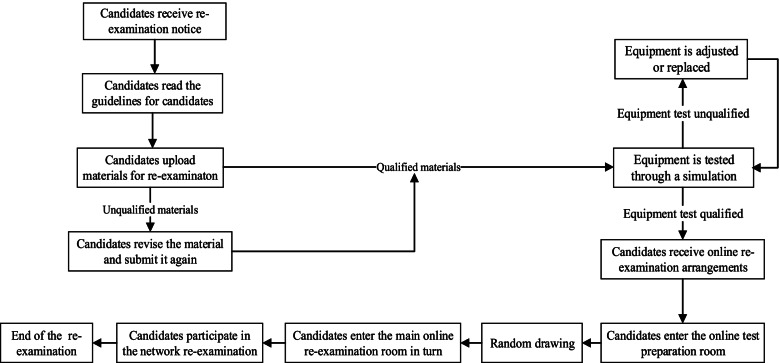
Flow chart of the online re-examination in PUTH

We used Cisco Webex Meetings (Version 40.7.1, 2020. Cisco Systems, Inc) for online re-examination. According to the requirements of the PKUHSC, the admission-application-ratio for online re-examination was 1:1.2, and PUTH determined the list of candidates for re-examination according to their preliminary scores, ranking them from high to low. The candidates had to complete the same test content as that of the on-site re-examination. The total score was calculated based on the score of the preliminary examination and the score of the re-examination, each accounting for 50%, and the final admission decision was made according to the total score. Figure 2 is the flow chart of the online re-examination in PUTH.

The candidates were required to download and install the Webex system in advance and prepare two seats, one seat for interviews and the other for monitoring of the site where the examinee was located. The candidates were required to have their video turned on at all times to allow real-time monitoring and prevention of cheating. A high-traffic network was used, and several simulation exercises of the whole process and all-round functional tests were performed, including tests of the clarity of the voice, video and screen sharing test questions of the ability of each candidate to participate in the formal re-examination securely and smoothly. The staff used the Webex platform to set up a network conference room as a test preparation room. The re-examination candidates were required to enter the test preparation room at the specified time. The staff members ensured that the candidates had the appropriate certificates; that they had the appropriate electronic equipment, including earphones; and that the room where the candidates were located met the test requirements. Facial recognition technology which connect the registration database with the public security platform was used to strengthen the verification of the candidates’ identities, and the staff read the test rules and performed a random drawing to determine the order in which the candidates would complete the re-examination. The candidates entered the main examination room one by one according to the randomly drawn order. Each re-examination, including the tests of comprehensive quality, English listening and speaking, professional knowledge, clinical skills and scientific research skills, etc., was scored by 5 examiners independently. The examination time for each component was 5-8 min, and the examination time of each examinee was more than 30 min. The comprehensive quality of the medical students, including their professional skills, communication ability and team work ability, was comprehensively evaluated. The re-examination score was the average of the five examiners’ scores.

Due to the COVID-19 pandemic, the requirements for online re-examination should conform to the principles of epidemic prevention and control. Therefore, arrangements were made to separate the participants; the density of personnel was reduced; personnel were prevented from gathering; and the identity check, site arrangement, the organization of facilities and equipment, and disinfection of work equipment were performed well ahead of time. It was ensured that the distance between the personnel at the re-examination location was more than 1 m.

### Statistical analysis

The data were managed by Excel 2019, and SPSS 25.0 was used for statistical analysis. Considering that the difficulty of the exam content might vary from year to year, the scores from different years were standardized on a scale from 0 to 100 to facilitate comparability among the years. The continuous data including age and scores conforming to the normal distribution are described as the mean ± standard deviation, and the independent sample t test was used for comparisons between groups. The continuous data not conforming to the normal distribution are described as the median (p25, p75), and the Mann-Whitney U test was used for comparisons between groups. The categorical data including gender, recent graduation, cultivation type and whether graduate from a key university, whether first choice and admission are described as numbers (percentages), and comparisons between groups were performed by the chi-square test. Multivariate analysis was performed by unconditional logistic regression. In all tests, statistical significance was set at two-sided *P* values less than 0.05.

## Results

### Basic information of the subjects

A total of 281 subjects were included in this study, of which 219 (77.9%) completed an on-site re-examination in 2017-2019 and 62 (22.1%) completed the 2020 online re-examination. The subjects included 135 males (48.0%) with an average age of 23.9±1.51 years old and 146 females (52.0%) with an average age of 23.7±1.55 years old. There was no statistically significant difference between the students who completed on-site and online re-examinations in terms of gender, recent graduation, cultivation type, graduate from a key university, and admission (*P*>0.05, Table 1). The students who participated in the online re-examination were slightly younger than the students who participated in an on-site re-examination, and the difference was statistically significant (*P*<0.05, Table 1).

There were statistically significant differences between the two groups of students in the preliminary test scores and the re-examination scores. These differences might have been related to the difficulty of the test papers in different years according to the overall enrollment and enrollment quota allocation. Therefore, the subsequent analyses based on the student ranking and the annual rankings were standardized by year.

**Table 1:** Baseline student characteristics in the two groups

Characteristic	On-site Re-examination (*n* = 219)	Online Re-examination (*n* = 62)	x^2^/t	*P*
Year			281.000	<0.001
2017	69(31.51)	0(0.00)		
2018	80(36.53)	0(0.00)		
2019	70(31.96)	0(0.00)		
2020	0(0.00)	62(100.00)		
Age (years)	23.98±1.62	23.63±0.94	2.144	0.033
Gender			0.122	0.727
Male	104(47.49)	31(50.00)		
Female	115(52.51)	31(50.00)		
Recent graduation			0.120	0.73
No	65(29.68)	17(27.42)		
Yes	154(70.32)	45(72.58)		
Cultivation type			0.958	0.328
Academic	67(30.60)	15(24.19)		
Professional	152(69.40)	47(75.81)		
Graduate from a key university			0.502	0.479
Yes	81(37.00)	26(41.92)		
No	138(63.00)	36(58.08)		
Admission			0.277	0.599
No	64(29.22)	16(25.81)		
Yes	155(70.78)	46(74.19)		
Subject 1 score	64.51±5.02	68.95±4.8	6.212	<0.001
Subject 2 score	68.21±7.6	72.5±6.74	4.020	<0.001
Comprehensive medicine score	226.69±16.96	237.44±13.7	4.579	<0.001
Re-examination Score	70.79±7.87	76.32±8.43	4.805	<0.001

### Analysis of the characteristics of the admitted students

In the on-site re-examinations, 70.8% of the students were admitted (155/219), and in the online re-examination, 74.2% of the students were admitted (46/62). Among the admitted students, the on-site and online re-examination results were very similar. There were no statistically significant differences in the individual characteristics (age, gender, etc.), preliminary test scores or re-examination scores among the admitted students (Table 2).

**Table 2:** Characteristics of the admitted students who completed an on-site re-examination or online re-examination

Characteristic	On-site Re-examination (*n*=155)	Online Re-examination (*n*=46)	t/x^2^	*P*
Age (years)	23.81±1.61	23.75±1.00	0.989	0.324
Gender			0.244	0.621
Male	88(56.77)	28(60.87)		
Female	67(43.23)	18(39.13)		
Recent graduation			0.580	0.446
No	46(29.68)	11(23.91)		
Yes	109(70.32)	35(76.09)		
Cultivation type			0.056	0.812
Academic	94(60.65)	27(58.70)		
Professional	61(39.35)	19(41.30)		
PUTH as the first choice			1.020	0.312
Yes	117(75.48)	38(82.61)		
No	38(24.52)	8(17.39)		
Standardized rank in Subject 1	56.52(29.71, 75.36)	53.23(29.03, 79.84)	-0.512	0.608
Standardized rank in Subject 2	56.88(31.88, 81.16)	56.85(32.26, 79.03)	0.287	0.774
Standardized rank in medicine	59.42(30.43, 80)	54.03(22.58, 75.81)	-0.904	0.366
Standardized rank of the re-examination score	65(45, 82.86)	64.11(43.55, 83.06)	-0.144	0.885

### Multivariable analysis of factors affecting admission

To explore the impact of online re-examination on student admissions, we conducted a multivariable logistic regression. Admission was the dependent variable, and the re-examination method, age, gender, recent graduation, PUTH as the first choice, graduate from a key university, cultivation type, standardized rank in Subject 1, standardized rank in Subject 2, standardized rank in medicine, and standardized rank of the re-examination score were the independent variables. The analysis results showed that online re-examination had no effect on student admissions (OR=2.465, 95% CI=0.738-8.231, *P*=0.142). Students seeking professional degree were less likely to be admitted than those seeking academic degree (OR=0.151, 95% CI=0.048-0.478, *P*=0.001), and those with a better standardized rank in medicine (OR=1.021, 95% CI=1.004-1.039, *P*=0.017) and those with a better standardized rank of the re-examination score (OR=1.126, 95% CI=1.089-1.164, *P*<0.001) were more likely to be admitted (Table 3).

**Table 3:** Multivariable analysis of factors affecting admission

Variable	Beta	*P*	OR	95% CI
Group				
On-site re-examination			1.000	
Online re-examination	0.902	0.142	2.465	0.738-8.231
Age	-0.058	0.768	0.944	0.642-1.388
Gender				
Female			1.000	
Male	-0.566	0.223	0.568	0.229-1.410
Recent graduation				
No			1.000	
Yes	-0.125	0.846	0.882	0.249-3.126
PUTH as the first choice				
Yes			1.000	
No	1.103	0.078	3.013	0.885-10.26
Graduate from a key university				
No			1.000	
Yes	-0.432	0.394	0.649	0.240-1.753
Cultivation type				
Academic			1.000	
Professional	-1.89	0.001	0.151	0.048-0.478
Standardized rank in Subject 1	0.002	0.828	1.002	0.985-1.019
Standardized rank in Subject 2	0.009	0.316	1.009	0.992-1.026
Standardized rank in medicine	0.021	0.017	1.021	1.004-1.039
Standardized rank of the re-examination score	0.118	0.000	1.126	1.089-1.164
Constant	-0.054	0.992	0.948	

## Discussion

In today’s age of technology, the worldwide exchange of information is conducted primarily through the Internet [19]. Because of the COVID-19 pandemic and the resulting social distancing policies, on-site re-examinations were suspended for 2020 postgraduate admissions in many medical schools in China. Due to the influence of COVID-19, the use of the Internet has been increasing gradually in teaching, training and assessment. Compared with the on-site re-examination format, the online re-examination format can save candidates’ time, travel costs and accommodation expenses, as reported in the literature [15]. If candidates take part in the traditional on-site re-examination, they have to spend a considerable amount of time, energy and financial resources on transportation and accommodation during the re-examination. Online re-examination could greatly reduces examinees’ round-trip time and expenses. However, there have been very few published reports on the application of online technology in postgraduate re-examination. It was expected that a virtual interview process would facilitate the re-examination and ease concerns about COVID-19 transmission.

As a retrospective study, comparability among different years were considered first in the design of this study since the data were from different temporal context. First, as a national major examination, the Unified National Graduate Entrance Examination is the selection test organized by the Ministry of Education of the People’s Republic of China under consistent principles and organizations in recent years. Second, as Table 1 shows, there was no statistically significant difference between the students who completed an on-site re-examination and those who completed an online re-examination in terms of gender, recent graduation, cultivation type, graduate from a key university, and admission (*P*>0.05). Moreover, as Table 2 shows, among the admitted students, the on-site and online re-examination results were very similar. There were no statistically significant differences in preliminary test scores or re-examination scores among the admitted students. Both baseline and admitted results suggested that the comparability may be spared from potential temporal biases. Therefore, although the format of online re-examination is not the same as that of on-site re-examination, online re-examination can fulfill the same admission criteria as on-site re-examination in the selection of medical postgraduates.

Considering that there may be fluctuations in test difficulty from year to year according to the overall enrollment and enrollment quota allocation, analyses based on the student ranking and the annual rankings were thus standardized by year. The results showed a higher score for online re-examination group in 2020. This may be due to several reasons including difficulty adjustment for the nationwide expansion of enrollment. Furthermore, as Table 3 shows, online re-examination had no effect on student admissions (OR=2.465, 95% CI=0.738-8.231, *P*=0.142). This finding also indicated that the online re-examination could be as objective and fair as on-site re-examination.

There are several measures to ensure the selective effect of online re-examination. (1) Since the organization process and implementation steps of online re-examination are new to the test administrators, examiners and candidates, a large number of simulation tests should be carried out in advance to ensure the successful completion of the online re-examination. In this study, 13 simulation tests were organized in advance, and 7 formal retests were conducted. The simulation test process covered the steps of the candidate entering the re-examination preparation room for his or her certificate to be verified, accessing the test room with two seats, participating in a random drawing to determine the re-examination order, and receiving instructions on matters requiring attention on the test, and then a simulation was performed to determine whether the audio, video and screen sharing of the main test room were functioning properly after the candidates logged in to the main examination room. It was found that 1/3 of the candidates were not proficient in operating the network system, and 1/5 of the candidates had unstable network connections. Therefore, the candidates were required to change their selected wireless network to a cable network. In addition, the candidates were required to prepare other backup equipment in advance to avoid equipment problems during the examination. After the simulation test drill, when the candidates participated in the formal re-examination, the re-examination process was very smooth. (2) Although the current online retest system required candidates to have two seats and their video turned on at all times, it was still difficult to achieve real-time cheating prevention. Therefore, it was very demanding to adjust the written test content, such as the professional knowledge and professional English content, to online re-examination. Distinct test questions were designed under national standard in order to further prevent leak of test questions. And the homogeneity between test papers were checked by an expert panel to ensure the fairness. In addition, in the process of organizing the online re-examination, we provided the supervisors with standardized question templates to ensure the standardized, structured content and unified difficulty coefficient for each examinee during the re-examination and to ensure the fairness, impartiality and objectivity of the online re-examination. (3) The time allotted for each component of assessment content need to be strictly controlled, and to do so, it was necessary to carry out examiner training and examinee simulation tests. Online re-examination involves greater technical requirements than on-site re-examination, and technical support personnel are required to help manage any technical problems in the virtual waiting room and the virtual test room. With the help of well-trained staff and high-speed Internet connections, the online re-examination was successfully completed.

Last but not the least, the present study analyzed factors affecting medical graduate admissions. Students seeking professional degree were less likely to be admitted than those seeking academic degree (OR=0.151, 95% CI=0.048-0.478, *P*=0.001), and those with better standardized ranks in medicine (OR=1.021, 95% CI=1.004-1.039, *P*=0.017) and those with better standardized ranks of the re-examination score (OR=1.126, 95% CI=1.089-1.164, *P*<0.001) were more likely to be admitted. As the number of graduate students registering for professional degree was higher than that registering for academic degree each year, the competition was more intense for professional degree, and the proportion of admitted students was lower. The re-examination, whether on-site or online is a selective examination which not only pay attention to students’ basic knowledge but also appreciate comprehensive quality and ability.

Limitations to the present online re-examination design are that the Internet connectivity speed or bandwidth may play an important role, especially for students overseas or in areas with poor connections. We found Internet connectivity to be an issue at times, but a repeated Internet connection test was prepared 1 week before the formal examination to ensure that all candidates’ problems would be solved before the online re-examination. In addition, examiners pointed out that only the candidates’ faces and upper bodies could be viewed due to the candidates’ sitting position. For this reason, we required candidates to stand in front of the camera and rotate 360 degrees during the re-examination. It allowed us to evaluate the overall appearance of the candidates, ensuring that there are no unrelated items or people in the test room such as books, written materials, other electronic equipments or people other than the candidate. Candidates were also required to sign a integrity commitment. In the near future, the use of a combination of virtual reality technology and artificial intelligence in the online re-examination process for the assessment of various candidate skills will continue to be explored. Finally, as affiliated hospital of Peking University, the present study has representativeness and influence in Chinese medical colleges, but as a single-center study, extrapolation may be limited.

## Conclusions

The present study showed that online re-examination could achieve same selective effect as on-site re-examination in the admission process for medical graduate students. It shed light on the feasibility of implementing online method for medical postgraduate enrollment during the COVID-19 pandemic. Remaining challenges include cheating prevention and the virtual assessment of clinical skills and laboratory skills, which need to be further explored.

## Data Availability

All data generated or analysed during this study are included in this published article.

## Abbreviations

PUTH: Peking University Third Hospital
SARS-CoV-2: severe acute respiratory syndrome coronavirus 2
2019-nCoV: 2019 novel coronavirus
COVID-19: coronavirus disease 2019
PKUHSC: Peking University Health Science Centre
